# Supplementary material for: Adoption of mobile learning in the university context: Systematic literature review
Source: PLoS One. 2024 Jun 7;19(6):e0304116. doi: 10.1371/journal.pone.0304116 (PMC11161115; doi:10.1371/journal.pone.0304116)
Supplement: Attachment — Submitted filename: Response letter.pdf [file pone.0304116.s002.pdf]

March 26th, 2024

Dear
Plos One

Kind regards

In accordance with the suggestions of the reviewers in our article "**Adoption of mobile learning in the university context: systematic literature review"**, the following changes weremade, properly marked with red letters in the article:

Reviewer	Comment	Response
General	(Figure 2) supply a replacement figure that complies with the CC BY 4.0 license. Please check copyright information on all replacement figures and update the figure caption with source information. If applicable, please specify in the figure caption text when a figure is similar but not identical to the original image and is therefore for illustrative purposes only. The following resources for replacing copyrighted map figures may be helpful:<br><br>USGS National Map Viewer (public domain): http://viewer.nationalmap.gov/viewer/<br>The Gateway to Astronaut Photography of Earth (public domain): http://eol.jsc.nasa.gov/sseop/clickmap/<br>Maps at the CIA (public domain): https://www.cia.gov/library/publications/the-world-factbook/index.html and https://www.cia.gov/library/publications/cia-maps-publications/index.html<br>NASA Earth Observatory (public domain): http://earthobservatory.nasa.gov/<br>Landsat: http://landsat.visibleearth.nasa.gov/<br>USGS EROS (Earth Resources Observatory and Science (EROS) Center) (public domain): http://eros.usgs.gov/#<br>Natural Earth (public domain): http://www.naturalearthdata.com/	The background map in Figure 2 is replaced with a map available in a copyright-free repository, as recommended by the editorial committee.
Reviewer 1	In the abstract section, it would be valuable to include a sentence discussing the implications of identified reseaech trends and patterns, as well as a brief acknowledgement of the study limitations to provide transparency.	The abstract is complemented based on what was suggested by the reviewer, mentioning the implications and briefly acknowledging the limitations, without exceeding the maximum number of words required by the journal.

Reviewer 1	I think the authors have done a good job. However, I think they should clearly show the reader how their work differs from others in terms of stating the paper's innovation and contribution.	Two additional paragraphs are added at the end of the introduction section, after the objective and questions, where the importance and contribution of this study is contextualized in light of its added value to the scientific literature.
Reviewer 2	Thank you very much. This is an interesting paper. However, there are few concerns that need to be addressed. 1) it is important to highlight the main contributions of this paper at the introduction section,	Two additional paragraphs are added at the end of the introduction section, after the objective and questions, where the importance and contribution of this study is contextualized in light of its added value to the scientific literature.
Reviewer 2	2) The study provides valuable insights into the adoption of mobile learning in university education, shedding light on both acceptance and rejection factors among students. However, there is a need for a deeper exploration of the various factors influencing the adoption and usage of these technologies. A more comprehensive analysis of these aspects would contribute significantly to optimizing mobile learning strategies and enhancing the overall educational experience.	Four new paragraphs are added to further explore the exploration of the main variables that contribute to the adoption of mobile learning, according to the reviewer.
Reviewer 2	3) The conclusions drawn from the reviewed literature effectively summarize the observed trends and patterns while also identifying crucial research gaps. These findings serve as a robust foundation for future research endeavors, emphasizing the significance of addressing these gaps in the current landscape of digital education. Further exploration of these research gaps is essential to advancing our understanding of mobile learning adoption dynamics.	Four additional paragraphs are added to expand on these identified gaps, as well as the importance of closing them for the scientific literature in general.
Reviewer 2	4) Additional studies should be included. This includes but not limited: - Solving the mystery of mobile learning adoption in higher education. doi: https://doi.org/10.1504/IJMC.2018.088271 - Modeling Students' Readiness to Adopt Mobile Learning in Higher Education: An Empirical Study. doi: https://doi.org/10.19173/irrodl.v19i1.3256	These important contributions are added to the analysis of the main mobile learning adoption variables, so that they are considered when interpreting these factors in the topic.

Reviewer 3	However, the methodology proposed has some elements that are not entirely clear. For example, I believe it would be interesting to explain why only two databases have been included. Why was ERIC not included, considering it is one of the main databases in education? Also, although the PRISMA methodology is mentioned for data selection, I would like to know more about the selection process.	In accordance with the reviewer's suggestions, an additional paragraph of limitations is added where it is mentioned that a database as important as ERIC was not included and that, consequently, future studies should do so. Additionally, a paragraph for clarity regarding the selection process was also added.
Reviewer 3	Regarding the results, they are relevant. However, I missed a more quantitative section summarizing the overall view of the studies in the sub-sections. This way, it could complement the qualitative information presented with a quantitative overview of the studies.	For this specific comment, it is considered that all the quantitative elements were presented graphically in the results section, while the discussion section simply expanded and delved into these quantitative results, so its characteristic is predominantly qualitative.
Reviewer 3	In line with this, the figures are relevant in the selected sections. However, their quality is somewhat low. It would be interesting to avoid pixelation upon enlargement by improving the resolution.	The figures are added in the highest quality available, improving the resolution

We look forward to your comments and hope to hear from you soon.

Thank you very much

–
The authors
